# Epidemiology of Traumatic Cervical Spinal Cord Injury in Southeast Norway

**DOI:** 10.1089/neur.2025.0013

**Published:** 2025-06-16

**Authors:** Mona Strøm, Jalal Mirzamohammadi, Thomas Glott, Tor Brommeland, Hege Linnerud, Pål Andre Rønning, Syed Ali Mujtaba Rizvi, Donata Biernat, Tor Arnøy Austad, Marianne Efskind Harr, Mads Aarhus, Eirik Helseth

**Affiliations:** ^1^ Spinal Unit, Sunnaas Rehabilitation Hospital, Nesodden, Norway.; ^2^ Department of Neurosurgery, Oslo University Hospital, Oslo, Norway.; ^3^ Department of Neuroradiology, Oslo University Hospital, Oslo, Norway.; ^4^ Institute of Clinical Medicine, Faculty of Medicine, University of Oslo, Oslo, Norway.

**Keywords:** comorbidity, epidemiology, injury prevention, Norway, spinal cord injuries, trauma mechanism

## Abstract

A traumatic cervical spinal cord injury (cSCI) is a severe consequence of trauma to the cervical spine with high mortality and morbidity rates. Epidemiological studies of traumatic cSCIs are necessary for planning preventive measures and health care resource allocation. This is a retrospective database study of 387 consecutive patients with traumatic cSCI admitted to hospitals in Southeast Norway between 2015 and 2022. The estimated incidence of traumatic cSCI was 1.6 per 100,000 per year. The incidence rates adjusted for standard European and global populations were 1.7 and 1.1 per 100,000 per year, respectively. The median patient age was 64 years, 75% were males, 40% had severe comorbidities, 65% of injuries were caused by falls, 25% were ethanol influenced, 44% had multiple traumas, and 96% were admitted to the Neurotrauma Center (NTC). In patients with C0–C2 injury, an odontoid fracture with dislocation of the odontoid fragment was most frequent. The most frequent subaxial injuries were, according to the AO Spine subaxial cervical spine injury classification system, minor nonstructural injuries (type A0) and translational injuries (type C). Eleven percent of patients were diagnosed with cSCIs at C0–C2, and 89% of cSCIs were subaxial. According to the American Spinal Injury Association (ASIA) Impairment Scale (AIS), 17% of cSCIs were classified as A, 12% B, 24% C, and 47% D. Forty-three percent of patients were classified as central cord syndrome, which was significantly associated with subaxial injuries and preinjury degenerative cervical spinal stenosis. Compromised respiration due to the cSCI itself was diagnosed in 17% of patients and was predominant in patients with complete cSCIs (AIS A or B) and high cervical injuries. These data will be helpful in planning the capacity of NTCs in the future. Interventions to prevent falls in elderly individuals and to increase awareness of ethanol as a risk factor for severe cSCIs are needed.

## Introduction 

A traumatic cervical spinal cord injury (cSCI) represents a severe manifestation of cervical spinal trauma and is associated with high rates of mortality and lifelong morbidity. The reported incidence of traumatic cSCIs in Europe is 0.5–2.6/100,000.^1–5^ The pooled incidence of traumatic cSCIs in developing countries, on the basis of a meta-analysis of 47 studies, is 1.0/100,000.^6^ The optimal patient outcome after cSCI depends on adequate acute care and rehabilitation.^7–13^

Historically, most cases of cSCI involve young men and traffic accidents, resulting in paresis of the arms and legs below the injury level.^14^ According to recent reports, an increasing fraction of patients with traumatic cSCI are elderly men and women with severe comorbidities sustaining a low-energy trauma, similar to what has been observed for patients with a traumatic brain injury.^15–18^ Many of these elderly patients present with central cord syndrome (CCS), a subtype of cSCI with more pronounced paresis in the arms than in the legs.^19–21^ In many patients with CCS, preinjury degenerative cervical stenosis makes the spinal cord vulnerable to even minor movements between vertebrae.^22,23^

The total care burden of patients with a cSCI depends on several factors: patient age and comorbidities, concomitant multiple trauma, type–level–grade of the cSCI, need for acute management at the Neurotrauma Center (NTC), need for intensive care unit (ICU) referrals, need for cervical surgical decompression/stabilization, and need for rehabilitation. Epidemiological studies of traumatic cSCIs are necessary for planning preventive measures and health care resource allocation.

We present epidemiological data on traumatic cSCIs in Southeast Norway, a region with a population of 3.1 million people from 2015 to 2022. The key issues were the incidence rate of cSCI, age and comorbidities of patients, injury mechanism, seasonal variations, type and level of the cSCI, and NTC referral rate.

We hypothesized that cSCI would be most common in higher age, falls would be the most common cause of injury, seasonal variation would be minimal, CCS would be common, and 100% of patients would be referred to an NTC for acute management.

## Materials and Methods

Oslo University Hospital (OUH) is the only NTC in Southeast Norway. Located in Oslo, this NTC serves all 20 local hospitals (LH). All trauma-related cervical procedures in this population are performed at OUH. The population within this health region increased from 2.9 million in 2015 to 3.1 million in 2022. A detailed description of the sex and age distribution of the Norwegian population can be found at www.ssb.no. This is a retrospective database study (with prospectively collected data) of all consecutive patients with cSCI included in our quality control database for traumatic cervical spine injuries (tCSIs) in Southeast Norway from January 1, 2015, to December 31, 2022. In the database, we prospectively registered all patients with tCSI C0/C1 to C7/Th1 diagnosed with cervical computed tomography (CT) and/or cervical magnetic resonance imaging (MRI) in Southeast Norway.^9,18,24^

The data retrieved from the database included time of injury, age, sex, admission to the NTC, trauma mechanism, ethanol influence at the time of injury, living status, and preinjury health status. Patients were classified according to the American Society of Anesthesiologists Physical Status Classification system (ASA score),^17^ ankylosing spondylitis, diffuse idiopathic skeletal hyperostosis (DISH), cervical spinal stenosis, cervical level of the SCI, and completeness of the lesion classified according to the American Spinal Injury Association (ASIA) Impairment Scale (AIS).^25^

CCS was defined as a cSCI resulting in more pronounced paresis in the arms than in the legs.^19,20^ The rates of compromised respiration due to the cSCI and the need for ventilation and tracheostomy were recorded.

The CSI causing the cSCI was for subaxial injuries classified according to the AO Spine subaxial cervical spine injury classification system,^26^ whereas the upper cervical injuries (C0–C2) were grouped as odontoid fractures, hangman fractures, or major discoligamentous complex injuries with distraction/dislocation in the C0–C2 region. Preinjury cervical spinal stenosis as the major finding on imaging was classified as A0, a minor nonstructural injury, according to the AO spine classification system.

Concomitant head injuries were scored according to the head injury severity score (HISS), and cases of multiple trauma were recorded.^18,27^

Data were summarized using frequencies for categorical data and median values for continuous data. Chi-square tests were used to compare categorical variables, and binary logistic regression analyses were used to investigate the effects of different covariates on CCS and compromised respiration due to traumatic cSCI. *p*-Values <0.05 were considered significant. The incidence per 100,000 people was calculated in person-years. For age-adjusted incidence according to the direct method, we used the 2013 European Standard Population (ESP) and the 2000–2025 World Health Organization’s (WHO) World Standard Population. Statistical analyses were performed with IBM SPSS version 29.0.

The study was approved by the OUH Data Protection Officer (DPO approval no 23/28298). The need to obtain informed consent from patients was waived. The quality control database for traumatic CS-Fx in southeastern Norway is approved by the OUH Data Protection Officer (DPO approval no 2014/12304).

## Results

**FIG. 1.**
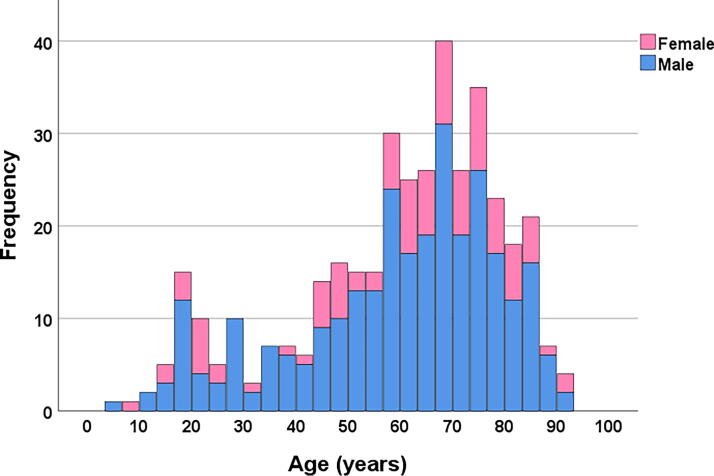
Number of patients with traumatic cervical spinal cord injury (cSCI) by age and sex (*n* = 387).

In the cohort population of Southeast Norway, we prospectively registered 3622 consecutive patients with tCSIs from 2015 to 2022. Concomitant cSCIs were observed in 387/3622 (10.7%) of patients, with an incidence of cSCI in Norway of 1.6/100,000. The age-adjusted incidence rates of cSCI in the ESP and the World Standard Population were 1.7/100,000 and 1.1/100,000, respectively. The median age of patients with a cSCI was 64 years (range 4.7–92.5 years, interquartile range 49.2–74.2 years), 75% were males, 49% were ≥65 years, and 44% had multiple traumas. Patient characteristics are presented in Table 1 and Table 2. Cervical SCIs occurred in all age-groups, with a small peak in patients aged 20–25 years and a major peak in patients aged 55–90 years (Fig. 1).

**Table 1.** Characteristics of Patients with Traumatic Cervical Spinal Cord Injuries (*n* = 387)

Variable	*N* (%)
Age	
≥65 years	190 (49.1)
Sex	
Male	289 (74.7)
Preinjury ASA score	
1–2 No/mild systemic disease	129 (59.2)
3 Severe systemic disease that is not a constant threat to life	143 (37)
4 Life-threatening systemic disease	13 (3.4)
Unknown	2 (0.4)
Living status at the time of injury	
Home—independent	342 (88.4)
Home—with assistance	31 (8)
Institutionalized	4 (1)
Unknown	10 (2.6)
Mechanism of injury	
Fall	252 (65.1)
Bicycle	48 (12.4)
4-wheel motor vehicle	21 (5.4)
Skiing	15 (3.9)
2-wheel motor vehicle	13 (3.4)
Diving	13 (3.4)
Sports, leisure activities, or play	12 (3.1)
Other	13 (3.4)
Under the influence of ethanol at the time of injury	98 (25.3)
Preinjury	
Cervical spinal stenosis	167 (43.2)
Ankylosing spondylitis	23 (5.9)
Diffuse idiopathic skeletal hyperostosis	9 (2.3)
Morphology of C0^a^–C2 injury	
Odontoid fracture	32 (8.3)
Hangman fracture	3 (0.8)
Major discoligamentous complex injury	6 (1.6)
Morphology of subaxial injury	
A0–Minor, nonstructural injury^b^	170 (43.9)
A1–Wedge-compression	6 (1.6)
A2–Split	7 (1.8)
A3–Incomplete burst	2 (0.5)
A4–Complete burst	12 (3.1)
B1–Posterior tension band injury (chance)	9 (2.3)
B2–Posterior tension band injury	6 (1.6)
B3–Anterior tension band injury	25 (6.5)
C–Translational injury	109 (28.2)
Type of cSCI	
Noncentral cord syndrome	220 (56.8)
Central cord syndrome	167 (43.2)
ASIA Impairment Scale (AIS)	
A No remaining motor or sensory function	65 (16.8)
B Sensory function, but no motor function, is preserved	48 (12.4)
C More than half of the key muscles below the neurological level have grade 3 or lower muscle strength	92 (23.8)
D At least half of the key muscles below the neurological level have grade 3 or higher muscle strength	182 (47)
Level of major spinal cord pathology on imaging	
C0–C2	37 (9.6)
C2/C3	14 (3.6)
C3/C4	59 (15.2)
C4/C5	95 (24.5)
C5/C6	106 (27.4)
C6/C7	69 (17.8)
C7/Th1	7 (1.8)
Multiple trauma	
Any	171 (44.2)
Admitted to neurotrauma center	370 (95.6)

^a^ C0—Occipital condyle.

^b^ A0 minor nonstructural injury—for 167/170 patients meaning pre-injury cervical stenosis.

ASA score, American Society of Anesthesiologists Physical Status Classification system score.

**Table 2.** Multiple Trauma in Patients with Traumatic Cervical Spinal Cord Injuries (*n* = 385, Two Cases with Missing Information Regarding Multiple Trauma)

Any multiple trauma	*N* (%)
Traumatic brain injury (TBI)	130 (33.6)
Mild	101 (26.1)
Moderate	13 (3.4)
Severe	16 (4.1)
Thoracolumbar fracture	43 (11.1)
Chest injury	51 (13.2)
Extremity fracture	29 (7.5)
Facial fracture	29 (7.5)
Abdominal injury	9 (2.3)
Pelvic injury	9 (2.3)

Severe preinjury comorbidities (ASA scores ≥3) were registered in 156/387 patients (40%), and preinjury dependent living was registered in 35/387 patients (9%). Thirteen patients (3%) had preinjury life-threatening systemic disease (ASA score = 4). Increasing age was associated with increased preinjury ASA scores and dependent living (*p* < 0.001).

**FIG. 2.**
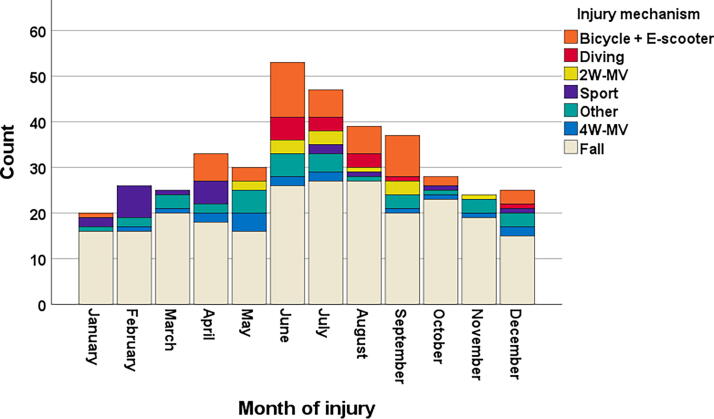
Number of patients with traumatic cervical spinal cord injury (cSCI) according to the month of injury and injury mechanism (*n* = 387).

During the 8-year study period, the annual number of patients with traumatic cSCI was stable, with a mean of 48 patients per year (range 42–53). However, there was clear seasonal variation, with a peak in cSCI from June to August, which is the summertime in Norway (Fig. 2). Injuries were significantly more common on Saturdays or Sundays than on weekdays (*p* < 0.001). The most common time of injury was the afternoon, followed by the evening, night, and morning (*p* < 0.001).

The most common injury mechanism was falls (65%), followed by bicycles (12%) and four-wheel motor vehicle accidents (5%; Table 1). The seasonal variation in number of patients with cSCI was associated with seasonal variations in injury mechanism (Fig. 2). Bicycle-, diving-, and two-wheel motor vehicle-related injuries were mainly observed during the summer (April–September). Fall injuries dominated during all seasons.

The influence of ethanol at the time of injury was recorded in 25% of the patients and was associated with injury during the evening and nighttime (*p* < 0.01). There was no significant sex difference in the influence of ethanol at the time of injury (25/98 females and 73/289 males, *p* = 0.142).

Cervical CT was performed on 387/387 (100%) patients, and cervical MRI was performed on 372/387 (96%) patients. The classification of the main CSI in the 387 patients with cSCIs is shown in Table 1. In patients with C0–C2 injury, an odontoid fracture with dislocation of the odontoid fragment was most frequent. In patients with subaxial injuries, minor nonstructural injuries (type A0) and translational injuries (type C) were most frequent. Preinjury ankylosing spondylitis or DISH in patients with subaxial injuries was observed in 23/346 and 9/346 patients, respectively.

**FIG. 3.**
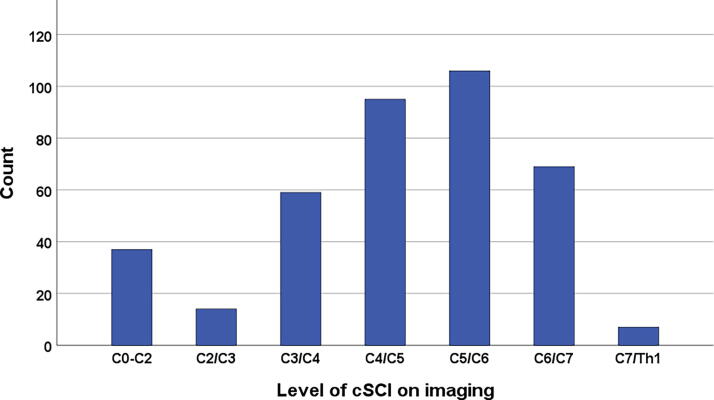
Number of patients with traumatic cervical spinal cord injury (cSCI) according to the level of major spinal cord pathology on imaging (*n* = 387).

Eleven percent of SCIs were at C0–C2, and 89% were subaxial (Table 1 and Fig. 3). An injury classification of CCS was found in 167/387 (43.2%) patients and non-CCS in 220/387 (56.8%) patients (Table 1). CCS was significantly associated with subaxial injuries compared with C0–C2 injuries (126/346 vs. 5/41, *p* = 0.026) and with degenerative cervical spinal stenosis compared with no stenosis (108/167 vs. 59/220, *p* < 0.001).

**FIG. 4.**
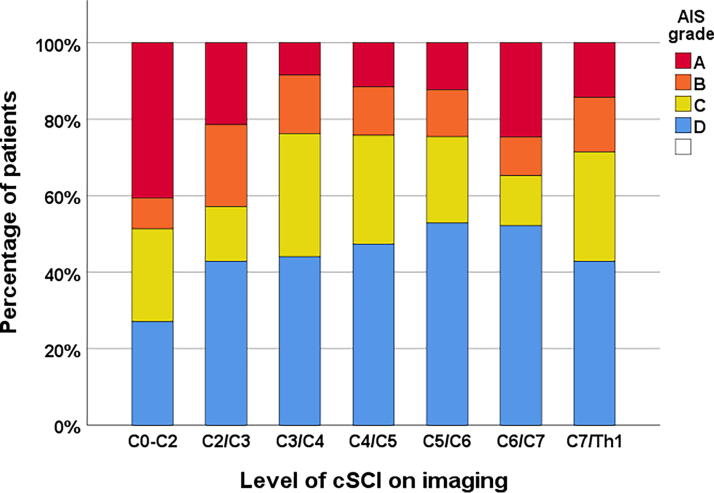
Level of major cervical spinal cord injury on imaging according to AIS grade (*n* = 387). AIS, American Spinal Injury Association (ASIA) Impairment Scale.

For AIS grade of the cSCI, see Table 1. Patients with CCS had either AIS grade D 133/167 (80%) or C 34/167 (20%), while AIS grades in patients with non-CCS were grade A in 65/220 (30%), B in 48/220 (22%), C in 58/220 (26%), and D in 49/220 (22%). Compared with Type A0 injuries, Type C injuries were more likely to be cSCI injuries with AIS grades of A and B (47/109 vs. 26/167, *p* < 0.001). AIS grade A or B SCIs were more common in upper cervical injuries than in lower cervical injuries (Fig. 4).

**FIG. 5.**
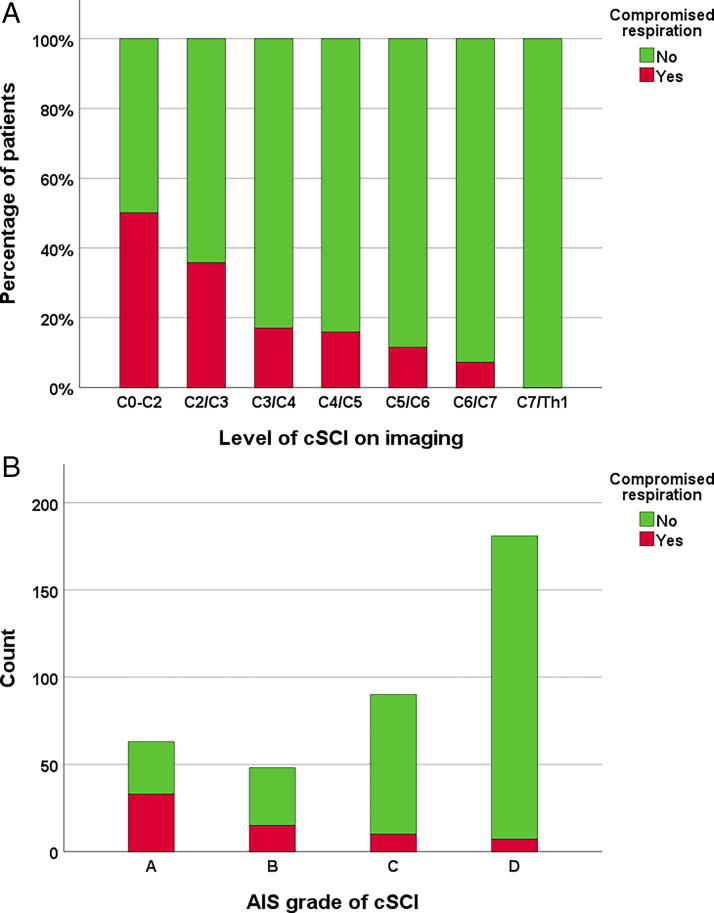
**(A)** Compromised respiration secondary to traumatic cervical spinal cord injury (cSCI) according to the level of the cSCI on imaging (*n* = 387). **(B)** Compromised respiration secondary to traumatic cervical spinal cord injury (cSCI) according to AIS grade (*n* = 387). AIS, American Spinal Injury Association (ASIA) Impairment Scale.

Compromised respiration due to the cSCI itself at admittance (not due to thoracic injury such as pneumothorax, flail chest, pulmonary contusion, or hemothorax) was diagnosed in 65/387 patients (16.8%). As expected, respiratory complications were predominant in patients with motor complete cSCIs (AIS A or B) above C4 (*p* < 0.001; Fig. 5). The number of patients in need of ventilator treatment after surgery during their stay at the NTC was 122/370 (33%), with a median length of ventilator treatment of 5 days (range 1–80 days). Tracheostomy during the NTC stay was performed in 49/370 (13%) patients.

Among the 387 patients with cSCIs diagnosed within our region during the study period, 370/387 (96%) were admitted to the NTC at OUH, 123/370 (33%) were directly transported to the NTC from the scene of the accident, and 247/370 (67%) were transferred from the LH after primary triage. Of the 17 patients who were not admitted to the NTC, 9 had sensory-only myelopathy or minimal paresis, 4 elderly patients had severe comorbidities, 2 patients had a potentially fatal C0-C2 injury, and 2 patients were admitted to the LH after acute treatment for a cSCI abroad.

## Discussion

In the studied cohort, we estimate the incidence of traumatic cSCI to be 1.6 per 100,000. There was a seasonal peak during the summer months, and 25% of the injuries were sustained under the influence of ethanol. A considerable proportion were elderly individuals with severe comorbidities and preinjury cervical spinal stenosis presenting with CCS after a fall injury. Almost all patients were transferred to the NTC for acute management.

### Incidence

In this study, we estimated the overall incidence of traumatic cSCI in the general population of Southeast Norway to be 1.6/100 000. This finding is in line with a recent population-based study from Italy in which the incidence of traumatic cSCI was estimated to be 1.4/100,000.^1^ Similar studies from Germany, Spain, Finland, and Denmark reported incidences of traumatic cSCI ranging from 0.5 to 2.6/100,000.^2–5^

However, the real incidence of traumatic cSCI in Norway is likely somewhat higher than that estimated in our study because of probable but undiagnosed and unreported cases of cSCI. The reasons for undiagnosed cases may be misdiagnosis due to subtle symptoms and clinical findings, trauma patients not seeking medical attention, and trauma victims who die at the scene of injury and are not transferred to the NTC. The number of patients with cSCI who were not transferred to the NTC from LH may have been underreported. It is mandatory for all Norwegian hospitals to report all inpatients and outpatients to the Norwegian Patients Registry with proper diagnosis and procedure codes to be reimbursed. A previous study from our group confirmed that more than 95% of CSIs were included in our registry and verified that the reporting from LH to NTC was very good.^28^

A previous Norwegian study conducted from 2012 to 2016 estimated the incidence of all levels of traumatic SCI in Norway to be 1.4/100,000, with 53% tetraplegia and 47% paraplegia, indicating an incidence of cSCI of ∼0.74/100,000.^29^ In this study, only patients who were transferred to specialized SCI rehabilitation departments were included.^30^ Rehabilitation-based studies are prone to coverage bias, as a large portion of patients with traumatic SCIs, for various reasons, are not referred to rehabilitation at specialized SCI departments.^31^

The incidence of many diseases is strongly associated with age, and adjusting for age allows for a more accurate comparison of disease incidence between populations with different age-groups. Age adjustment of the Norwegian cSCI incidence rates according to the ESP and the World Standard Population yielded incidences of 1.7/100,000 and 1.1/100,000, respectively. The age distribution in the Norwegian population is similar to that in Europe, while the world has a significantly younger population.

A systematic review on the incidence of traumatic SCI worldwide published in 2023 revealed very large variation in reported rates, and most of the studies were of low quality and lacked consistent case selection due to unclear definitions and unclear ascertainment methods.^32^ Several studies evaluating the global incidence of traumatic SCI indicate that the incidence of traumatic SCI is higher in low- and middle-income countries than in high-income countries,^33–36^ but traumatic SCIs have been insufficiently registered in many countries (high- and low-income), emphasizing the need for reliable population-based epidemiological studies.

### Patient characteristics

Most studies revealed a male predominance, which is consistent with the findings of our present study, which included 75% male patients.^1,4,35,37^ There are several potential reasons why males may be overrepresented. Males generally engage in more high-risk activities, such as sports or certain occupations, which increase their likelihood of traumatic injuries. Additionally, males tend to have a greater propensity for risk-taking behavior, which can further contribute to their involvement in traumatic incidents.^38^

Epidemiological studies on populations with SCIs revealed a bimodal age distribution, with a small increase in young adults/adolescents and a major increase among the elderly.^5,36^ This finding is consistent with our results, with a minor increase in patients aged 20–25 years and a significant increase in patients aged 55–90 years. As expected, almost half of the patients (49%) in our study were considered elderly individuals (age ≥65 years) according to the WHO definition of elderly, which is often associated with significant comorbidities. Elderly individuals constitute the greatest proportion of the traumatic brain injury population as well as the cSCI population.^15–17^ In recent decades, the number of traumatic SCIs has increased among elderly people.^2,35–37,39,40^ This increase is likely due to a significant rise in the total number of elderly individuals^41^ and a corresponding increase in fall-induced injuries as the general population ages. Elderly patients are more vulnerable to traumatic SCI due to several factors, including changes in bone quality related to aging (osteopenia, osteoporosis) and an increasing prevalence of degenerative cervical spinal stenosis.^22^ In addition, elderly patients have a higher risk of falls due to loss of sensory function (vision, the vestibular system, and proprioception) and greatly diminished postural control.^42,43^ The increased risk and occurrence of other medical conditions, neuromuscular and neurological disorders, and the use of medications causing postural instability can increase the risk of falls and subsequent SCI in elderly patients.^22,44,45^ The treatment of elderly patients with cSCI requires a comprehensive understanding of their particular pathophysiological characteristics, age-related disorders, and the associated comorbidities and frailty. According to the Norwegian prescription registry, half of the Norwegian population ≥65 years of age uses antithrombotic drugs, which can have an impact on the course of the injury and may increase the risk of surgery.^46^ Given their limited physiological reserves and preexisting medical comorbidities, elderly patients are considered to have a higher risk of morbidity and mortality from SCIs.^47^

### Trauma mechanism and seasonal variation

The injury mechanism of traumatic SCI differs between low-income and high-income countries. The World Bank classifies the world’s economies into four groups.^48^ In low- to middle-income countries, transport-related injuries are the leading cause of traumatic SCI, whereas in high-income countries, falls are the main etiology of traumatic SCI, especially among elderly individuals.^6,34,35,49^ The growing aging population in high-income countries indicates that traumatic SCI resulting from falls may become an increasing public health challenge.^35^ Therefore, understanding the epidemiology of traumatic SCI in different countries is important for planning cost-effective preventive measures.

In line with our hypothesis, the most common trauma mechanism for cSCI in our study was falls (65%). The high incidence of fall injuries most likely reflects the high median age of the patients. As the aging population grows, the number of fall-related injuries is expected to rise unless effective fall prevention strategies are implemented. Understanding the mechanisms of falls will be useful for future interventions to identify high-risk persons, activities, and environmental factors to prevent falls and fall-induced cSCI.^42,50–52^ Different prevention strategies should be provided and may include fall prevention education; advice on physical activity; medication review; strength and balance exercise; the management of different medical conditions (orthostatic hypotension, incontinence, foot problems or other acute/chronic conditions); the optimization of vision, hearing, and nutrition; and improvements in home safety.

In our study, bicycle accidents were the second most common cause of cSCI, occurring more frequently than MVAs. In recent years, the popularity of cycling for transport and recreation has increased. The growing number of bicyclists, particularly during rush-hour traffic, has led to an increase in bicycle-related injuries.^53^ Recently, measures to increase road safety for cyclists and awareness campaigns to reduce the risk of bicycle-related injuries in Norway have been implemented.^54^ MVA is a common cause of SCI in many countries, especially low-income countries.^6^ Compared with other nations, Norway stands out as a country with a relatively low incidence of MVAs, and in recent decades, road safety policies have successfully reduced the number of MVAs.^55^

The influence of ethanol has been correlated with an increased risk of SCI.^49^ When ethanol is consumed, it affects coordination, judgment, and reaction time, thus increasing the risk of accidents and injuries. In our study, 25% of patients were under the influence of ethanol at the time of injury, with an increased proportion of injuries during the evening and nighttime. Thus, the prevention of ethanol-related injuries requires a multifaceted approach that involves education, responsible ethanol service practices, awareness campaigns, and adequate enforcement of laws. By promoting responsible ethanol consumption, we can strive to reduce the incidence of ethanol-related injuries.^56^

The seasonal variation observed, with a peak of cSCIs during the summer months, could to some extent be explained by seasonal variations in the injury mechanism. Bicycle-, diving-, and 2W-MVA-related injuries were mainly observed during the summertime (April–September), whereas skiing injuries were mainly observed during the wintertime (October–March). Fall injuries dominated during all seasons. The peak of cSCI during the summer months coincides with the holiday period in Norway, where the capacity at acute care hospitals and rehabilitation centers is reduced. This can lead to potential delays in care for this patient group.

### Description of cSCI

The most frequent morphological injury in the upper cervical spine was C2 odontoid fx, which is the most common CS-Fx in the elderly and constitutes 20% of all CS-Fx in Norway.^18^ Concomitant cSCI is only observed in 4% of odontoid fractures.^57^ However, the number of patients with odontoid fractures and severe cSCI may be underreported/undiagnosed when the patient dies at the scene of the injury.

We found a clear preponderance of subaxial injuries compared with injuries at C0–C2 (89% vs. 11%). According to the AO Spine subaxial classification system,^26,58^ the two most common morphological injuries associated with traumatic cSCI are minor nonstructural injuries (Type A0 injuries) and translational injuries (Type C injuries). Type C injuries involve significant displacement or translation of vertebral segments with compression of the spinal cord, are usually associated with fracture and discoligamentous rupture, and are, by definition, unstable injuries. The typical type A0 injury is significant degenerative spinal canal stenosis, where we assume that a cSCI is caused by minor movement at the affected segment, often without significant fracture or rupture of ligaments.^18,22,28,57^ Type C injuries compared with Type A0 injuries had more often cSCI injuries with AIS grade A and B.

Schneider et al.^20^ first described clinical CCS, a clinical description of hyperextension trauma and considerably more weakness in the arms than in the legs. Several authors have reported an association between CCS and preinjury degenerative cervical spinal stenosis.^21,23,59^ In our study, 43% of the cSCIs were classified as CCS on the basis of typical clinical findings. We also found that CCS was more common subaxially and associated with degenerative cervical spinal stenosis. This finding confirms our hypothesis that CCS is common and associated with cervical spinal stenosis. Because the initial cervical CT images may not show fractures and clinical symptoms may be mild, the diagnosis of CCS may be delayed or overlooked.^60,61^

Preinjury ankylosing spondylitis (AS) was found in 6% of the patients with cSCI. AS is associated with an increased risk of CS-Fx and with cSCI.^56,62–64^ CS-Fxs in patients with AS are often subaxial and, according to the AO classification of subaxial fractures, categorized as B1-posterior tension band injury (bony) or as Type CB1 translational injuries.^19^ B1 and CB1 are unstable fractures.

The completeness of cSCI at admission was classified as AIS grade A in 17% of the patients, B in 12%, C in 24%, and D in 47%. Identifying degree of completeness during the acute phase can be difficult, as the patient’s condition can prevent a detailed neurological assessment. It is essential to acknowledge this limitation and consider it when evaluating the patient’s potential for neurological improvement.

Classification of neurological impairments according to the International Standards for Neurological and Functional Classification of Spinal Cord Injury Patients (International Standards) standardizes the assessment of the severity and level of the injury, and the prediction of outcome can be performed on the basis of parameters from the International Standards.^65^ Estimating long-term outcomes early after traumatic SCI is important for defining management strategies and providing patients and families with more precise information about long-term prognosis. If surviving the acute phase, many patients with traumatic cSCI will be in need of specialized rehabilitation to improve neurological outcomes and adapt their new functional level to ensure participation, activities, and quality of life.^10–12^

### Respiratory complications

In our study, compromised respiration due to cSCI was observed in 17% of the patients with cSCI and was much more common in those with C0–C2 injuries than in those with subaxial injuries. Other diseases and associated injuries (thoracic injury, such as pneumothorax, flail chest, lung contusion, or hemothorax) can significantly increase respiratory challenges during the acute phase and can explain the high number of patients in need of ventilator treatment (33%) and tracheostomy (13%) in our study. Respiratory dysfunction is a major cause of morbidity and mortality in SCI patients due to impairment of respiratory muscles, reduced vital capacity, ineffective cough, a reduction in lung and chest wall compliance, and excess oxygen costs associated with breathing due to distortion of the respiratory system.^66^ The development of respiratory complications is directly correlated with the level of injury and the degree of motor completeness, physiologically explained by the respiratory center at levels C0–C2, the phrenic nerve innervation at levels C3–C5, and the affected intercostal muscles. Prevention of respiratory complications needs to begin immediately after injury, and transfer to an SCI center specializing in acute management of tetraplegia has been shown to significantly reduce the number of respiratory complications.^67^

### Referral rate to the NTC

Among the 387 patients with traumatic cSCI during the study period, 96% were admitted to the NTC; 33% were admitted directly from accident site, and 67% were transferred from an LH after primary triage. Minimal neurological findings, elderly individuals with severe comorbidities, fatal injuries, and patients treated abroad and then transferred to LH are possible reasons why the referral rate to a NTC for acute treatment was not 100%, as was our hypothesis.

The key elements of acute care in the NTC are monitoring and treatment in the ICU and surgical cervical stabilization/decompression.^9,24^

### Strengths and limitations

This is a -based retrospective database study with prospectively collected data. In Norway, everyone has equal access, and the government reimburses the health care system for prehospital care, hospital care, and rehabilitation. The data can most likely be externally validated by studies conducted in countries with similar age distributions, trauma organizations, and trauma mechanism profiles.

The specific aim of this study was not planned when designing the database. Thus, the retrospective nature of the study is a potential weakness. As mentioned in the discussion, there are most likely some missed cases due to underreporting or missed diagnoses.

## Conclusions

The incidence of cSCI in Norway is 1.6 per 100,000. There was a seasonal peak during the summer months, and 25% of injuries were sustained under the influence of ethanol. A considerable proportion of the patients were elderly individuals with severe comorbidities and preinjury cervical spinal stenosis presenting with CCS after a fall injury. Almost all patients were transferred to the NTC for acute management. These data will be helpful in planning the capacity of NTCs in the future. Interventions should aim to prevent falls in elderly individuals and increase awareness about ethanol as a risk factor for severe injuries.

## Transparency, Rigor, and Reproducibility Summary

This study adheres to the highest standards of transparency, rigor, and reproducibility. All data analyses were conducted following best practices in the field of epidemiology. Detailed descriptions of the methodologies, including data collection and analysis, are provided to ensure that the study can be accurately replicated by other researchers. All raw data and relevant materials are available upon request.

## Data Sharing Statement

The data that support the findings of this study are available from the corresponding author upon reasonable request. Due to the sensitive nature of the data, access is restricted to ensure participant confidentiality. The data are stored securely at Department of Neurosurgery at OUH and will be made available to qualified researchers who meet the criteria for access to confidential data.
